# Supplementary material for: Reduced numbers of T cells and B cells correlates with persistent SARS-CoV-2 presence in non-severe COVID-19 patients
Source: Sci Rep. 2020 Oct 19;10:17718. doi: 10.1038/s41598-020-73955-8 (PMC7573596; doi:10.1038/s41598-020-73955-8)
Supplement: Supplementary file 1 — Supplementary Tables. [file 41598_2020_73955_MOESM1_ESM.docx]

**Persistent SARS-CoV-2 presence is companied with defects in adaptive immune system in non-severe COVID-19 patients**

# Running title: Persistent SARS-CoV-2  and immune shedding

Bing Liu MD; Department of Respiratory and Critical Care Medicine, Zhongnan Hospital of Wuhan University, Wuhan, China

Junyan Han PhD; Department of Immunology, School of Basic Medicine, Tongji Medical College, Huazhong University of Science and Technology, Wuhan, China

Xiaohuan Cheng MD; Department of Laboratory, Zhongnan Hospital of Wuhan University, Wuhan, China

Long Yu MS; Analytical and Testing Center, School of Basic Medicine, Tongji Medical College, Huazhong University of Science and Technology, Wuhan, China

Li Zhang MD, PhD; Department of Respiratory and Critical Care Medicine, Zhongnan Hospital of Wuhan University, Wuhan, China

Wei Wang MD, PhD; Department of Respiratory and Critical Care Medicine, Zhongnan Hospital of Wuhan University, Wuhan, China

Lan Ni MD, PhD; Department of Respiratory and Critical Care Medicine, Zhongnan Hospital of Wuhan University, Wuhan, China

Chaojie Wei MD, PhD; Department of Respiratory and Critical Care Medicine, Zhongnan Hospital of Wuhan University, Wuhan, China

Yafei Huang* MD, PhD; Department of Pathogen Biology, School of Basic Medicine, Tongji Medical College, Huazhong University of Science and Technology, Wuhan, China

Zhenshun Cheng* MD; Department of Respiratory Medicine, Zhongnan Hospital of Wuhan University, Wuhan, China

* Author Y.H. and Z.C. contributed equally to this manuscript

**Key words:** COVID-19, SARS-CoV-2, shedding, adaptive immune system, lymphocyte subpopulations.

**Corresponding author:**

Zhenshun Cheng MD; Department of Respiratory and Critical Care Medicine, Zhongnan Hospital of Wuhan University, Wuhan, China zhenshun_cheng@126.com

**Alternate corresponding author:**

Yafei Huang MD, PhD, Department of Pathogen Biology, School of Basic Medicine, Tongji Medical College, Huazhong University of Science and Technology, Wuhan, China huangy2018@hust.edu.cn

**Summary**

Defects in adaptive immune system, including reduced T cells and B cells, were frequently observed in non-severe COVID-19 patients with persistent SARS-CoV-2 shedding. Assessment of immune system could be clinically relevant for discharge management.

**Supplementary table 1 Baseline characteristics of PPP and PPN patients**

| **No. (%)** | **PPP (n=19)** | **PPN (n=18)** |  | ***P* value** |
| --- | --- | --- | --- | --- |
| **Symptoms** |  |  |  |  |
| Fever | 16 (84.2) | 13 (74.2) |  | 0.38 |
| Cough | 17(89.5) | 12(66.7) |  | 0.09 |
| Expectoration | 7(16.8) | 2(11.1) |  | 0.07 |
| Hemoptysis | 1 (5.3) | 0 (0) |  | 0.32 |
| Dyspnea | 3 (15.8) | 8 (44.4) |  | 0.06 |
| [Weep tears](https://fanyi.so.com/?src=onebox#shed%2Fweep%20tears) | 1 (5.3) | 0 (0) |  | 0.32 |
| Pharyngalgia | 0 (0) | 1 (5.3) |  | 0.30 |
| [Diarrhoea](https://fanyi.so.com/?src=onebox#diarrhoea) | 3 (15.8) | 2 (11.1) |  | 0.68 |
| **Comorbidities** |  |  |  |  |
| CVD | 3 (15.8) | 2 (11.1) |  | 0.68 |
| Diabetes | 2 (10.5) | 0 (0) |  | 0.16 |
| Hepatitis | 1 (5.3) | 1 (5.6) |  | 1 |
| Other | 0 (0) | 1 (5.6) |  | 0.30 |
| **Treatments** |  |  |  |  |
| GC | 0 (0) | 2 (11.1) |  | 0.14 |
| Antiviral | 15 (78.9) | 11 (61.1) |  | 0.23 |
| Antibiotics | 7 (36.8) | 10 (55.6) |  | 0.52 |
| TCM | 17 (89.5) | 13 (72.2) |  | 0.18 |
| **Others** |  |  |  |  |
| Smoke | 0 (0) | 3 (16.7) |  | 0.06 |
| Drink | 0 (0) | 3 (16.7) |  | 0.06 |
| Duration of SARS-CoV-2 shedding (days, median [IQR]) | 32 (30-33) | 33 (30-35) |  | 0.25 |

Data are median (IQR) or n (%). P values were obtained from χ² tests , Fisher’s exact tests, T tests or Mann–Whitney U tests, when appropriate. P <0.05 was considered statistically significant (in bold).

Abbreviations: COVID-19, coronavirus disease 19; PP (persistently positive); PPP, PP patients tested positive again; PPN: PP patients tested negative.

**Supplementary table 2 Laboratory testing results of PPP and PPN patients.**

| **Median (IQR)** | **Normal range** | **PPP (n=19)** | **PPN (n=18)** | ***P* Value** |
| --- | --- | --- | --- | --- |
| **Blood cells** |  |  |  |  |
| WBC (x10^9^/L) | 3.5-9.5 | 5.0 (4.6-5.6) | 4.9 (4.2-6.3) | 0.59 |
| Neutrophils (x10^9^/L) | 1.8-6.3 | 2.9 (2.3-3.2) | 2.7 (2.3-3.7) | 0.54 |
| Lymphocytes (x10^9^/L) | 1.1-3.2 | 1.6 (1.2-1.9) | 1.5 (1.4-1.7) | 0.84 |
| PLTs (x10^9^/L) | 125-350 | 185 (169-227) | 176 (148-212) | 0.68 |
| **Blood biochemicals** |  |  |  |  |
| Hb (g/L) | 130-175 | 138 (123-146) | 129 (116-140) | 0.31 |
| ALB (g/L) | 40-55 | 43.4 (41.7-44.8) | 40.2 (37.3-42.7) | 0.06 |
| ALT (U/L) | 9-50 | 26.0 (20.5-35.5) | 29.5 (18.5-47.3) | 0.76 |
| AST (U/L) | 15-40 | 23.0 (19.0-30.0) | 25.5 (14.3-32.5) | 0.99 |
| Total Bilirubin (μmol/L) | 5-21 | 12.9 (10.9-16.0) | 12.9 (9.5-16.6) |  |
| **Inflammatory biomarkers** |  |  |  |  |
| CRP (g/L) | 0-10 | 1.8 (0.9-2.4) | 1.9 (1.3-3.2) | 0.14 |
| SAA (mg/L) | 0-10 | 6.5 (4.5-10.1) | 6.2 (5.3-10.2) | 0.35 |
| IL-6 (pg/mL) | 0-7 | 2.2 (1.5-2.5) | 2.7 (1.8-4.7) | 0.06 |

Data are median (IQR) or n (%). P values were obtained from χ² tests , Fisher’s exact tests, T tests or Mann–Whitney U tests, when appropriate. P <0.05 was considered statistically significant (in bold).

Abbreviations: COVID-19, coronavirus disease 19; PP (persistently positive); PPP, PP patients tested positive again; PPN: PP patients tested negative; PLT, platelets; Hb, hemoglobin; ALB, albumin; ALT, alanine aminotransferase; AST, aspartate aminotransfease; CRP, C-reactive protein; SAA, serum amyloid A..
